# Mechanical Insights into Aggregation‐Induced Delayed Fluorescence Materials with Anti‐Kasha Behavior

**DOI:** 10.1002/advs.201801629

**Published:** 2018-12-07

**Authors:** Jingjing Guo, Jianzhong Fan, Lili Lin, Jiajie Zeng, Hao Liu, Chuan‐Kui Wang, Zujin Zhao, Ben Zhong Tang

**Affiliations:** ^1^ State Key Laboratory of Luminescent Materials and Devices Center for Aggregation‐Induced Emission South China University of Technology Guangzhou 510640 China; ^2^ Shandong Province Key Laboratory of Medical Physics and Image Processing Technology Institute of Materials and Clean Energy School of Physics and Electronics Shandong Normal University Jinan 250014 China; ^3^ Department of Chemistry Hong Kong Branch of Chinese National Engineering Research Center for Tissue Restoration and Reconstruction The Hong Kong University of Science & Technology Clear Water Bay Kowloon Hong Kong China

**Keywords:** aggregation‐induced delayed fluorescence, anti‐Kasha emission, internal conversion, intersystem crossing, organic light‐emitting diodes

## Abstract

Organic materials with aggregation‐induced delayed fluorescence (AIDF) have exhibited impressive merits for improving electroluminescence efficiency and decreasing efficiency roll‐off of nondoped organic light‐emitting diodes (OLEDs). However, the lack of comprehensive insights into the underlying mechanism may impede further development and application of AIDF materials. Herein, AIDF materials consisting of benzoyl serving as an electron acceptor, and phenoxazine and fluorene derivatives as electron donors are reported. They display greatly enhanced fluorescence with increased delayed component upon aggregate formation. Experimental and theoretical investigations reveal that this AIDF phenomenon can be rationally ascribed to the suppression of internal conversion and the promotion of intersystem crossing in solid. Moreover, the theoretical calculations disclose that the efficient solid‐state delayed fluorescence originates from the higher energy electronic excited state (e.g., S_2_) rather than the lowest energy‐excited state (S_1_), demonstrating an anti‐Kasha behavior. The excellent AIDF property allows high exciton utilization and thus superb performance of OLEDs using these new materials as light‐emitting layers.

## 1 Introduction

Organic light‐emitting diodes (OLEDs)^1^, ^2^ have gone through significant advances and achieved the stage of commercial applications. Nowadays, they are deemed to be the most promising candidates for new‐generation flexible displays and lighting sources, owing to the merits of self‐luminescence, flexibility, high efficiency, full‐color emission, and so forth. Excellent electroluminescence (EL) efficiency, high operational stability, and simple fabrication procedure have always been the critical factors for OLED commercialization. To produce cost‐effective OLEDs, high‐performance nondoped OLEDs are thus expected to be the ideal alternative to the current doping technique. However, most conventional phosphorescent materials or thermally activated delayed fluorescence (TADF) emitters that are able to fully harvest electrogenerated excitons suffer from concentration‐caused quenching, which greatly limits their practical application in nondoped OLEDs.^3^, ^4^, ^5^, ^6^, ^7^, ^8^

Recently, aggregation‐induced delayed fluorescence (AIDF) materials are attracting great interest because of their high exciton utilization, excellent neat‐film photoluminescence (PL) efficiency, and impressive advantages in the fabrication of highly efficient and stable nondoped OLEDs.^9^, ^10^, ^11^ Owing to their fundamental importance and practical implications, more and more researchers gradually embark on this fascinating research area, and a library of AIDF materials with various molecular structures and brilliant EL performances have been developed.^10^, ^11^, ^12^, ^13^, ^14^, ^15^, ^16^, ^17^, ^18^ Actually, AIDF is an interesting photophysical phenomenon associated with molecular aggregation, that is, fluorogenic molecules exhibit weak emission and negligible delayed fluorescence in solution but strong emission with prominent delayed components in aggregate or in neat film. However, its underlying mechanism has not been well decoded. So, filling the gap of knowledge in this area is of high academic importance for in‐depth understanding of fundamental photophysical processes and rational design of efficient luminescent materials.

**Figure 1:**
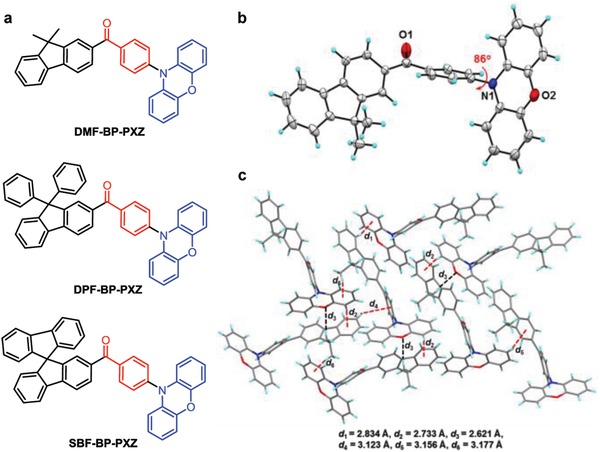
a) Molecular structures of DMF‐BP‐PXZ, DPF‐BP‐PXZ, and SBF‐BP‐PXZ, b) crystal structure (CCDC 1572670) and c) packing pattern of DMF‐BP‐PXZ in crystals.

To decipher the AIDF process, herein, we design and synthesize three new emitters (DMF‐BP‐PXZ, DPF‐BP‐PXZ, and SBF‐BP‐PXZ) that consist of electron‐donating phenoxazine (PXZ) and fluorene derivatives coupled with an electron‐withdrawing benzoyl core (**Figure** **1**a). All three molecules exhibit weak emissions without delayed fluorescence in solutions but strong emissions with obvious delayed components upon aggregate formation, behaving prominent AIDF feature. By combining the photophysical measurements and theoretical calculations, we demonstrate that this phenomenon can be rationally ascribed to the suppression of internal conversion (IC) channels and the promotion of intersystem crossing (ISC) processes in the aggregated state. And interestingly, the unusual anti‐Kasha behavior is observed for the materials, namely their emissions originate from the higher energy electronic excited state rather than the lowest one. Moreover, their EL behaviors are investigated in nondoped and doped OLEDs, which afford excellent EL efficiencies with negligible efficiency roll‐off over a broad brightness range.

## 2 Results and Discussion

### 2.1 Synthesis and Characterization

The new emitters DMF‐BP‐PXZ, DPF‐BP‐PXZ, and SBF‐BP‐PXZ were easily prepared according to the synthetic routes involving two‐steps reactions with high yields as described in Supporting Information. The key intermediates DMF‐BP‐Br, DPF‐BP‐Br, and SBF‐BP‐Br were synthesized using Friedel–Crafts acylations. Then, Buchwald–Hartwig aminations of the preprepared intermediates with the corresponding electron‐donating PXZ unit in the presence of Pd(OAc)_2_/P(t‐Bu)_3_/t‐BuONa afforded the target molecules. All final products were further purified by temperature‐gradient vacuum sublimation after column chromatography. The molecular structures were confirmed by NMR and high‐resolution mass spectrometry. They are highly soluble in common organic solvents including tetrahydrofuran (THF), dichloromethane, chloroform, toluene, etc., but insoluble in water and ethanol. The thermal properties of DMF‐BP‐PXZ, DPF‐BP‐PXZ, and SBF‐BP‐PXZ were investigated by means of thermogravimetric analysis and differential scanning calorimetry at a heating rate of 10 °C min^−1^ under nitrogen atmosphere. The high decomposition temperatures (*T*_d_, corresponding to 5% weight loss) of 341, 395, and 398 °C, and high glass‐transition temperatures (*T*_g_) of 83, 126, and 132 °C are recorded for DMF‐BP‐PXZ, DPF‐BP‐PXZ, and SBF‐BP‐PXZ, respectively (Figure S1, Supporting Information), which are conducive to fabricating stable EL devices. The electrochemical properties of DMF‐BP‐PXZ, DPF‐BP‐PXZ, and SBF‐BP‐PXZ in neat films were measured by cyclic voltammetry. Similar reversible oxidation and reduction processes are observed for these emitters (Figure S2, Supporting Information), indicating their good electrochemical stability. Based on the onset of oxidation and reduction potentials relative to Fc/Fc^+^, the highest occupied molecular orbital (HOMO) energy levels are estimated to be −5.01 eV for DMF‐BP‐PXZ, −5.02 eV for DPF‐BP‐PXZ, and −5.04 eV for SBF‐BP‐PXZ, respectively. The lowest unoccupied molecular orbitals (LUMO) energy levels of DMF‐BP‐PXZ, DPF‐BP‐PXZ, and SBF‐BP‐PXZ are −2.83, −2.84, and −2.88 eV, respectively.

### 2.2 Crystal Structures

Single crystals of DMF‐BP‐PXZ were grown from the mixture solvent system of THF/ethanol at room temperature, and the crystals structure is displayed in Figure 1b. The molecule adopts a highly twisted conformation, in which the PXZ moiety is nearly orthogonal to the adjacent phenylene substituent with a large torsion angle of 86°. This can effectively prevent close intermolecular π–π stacking, and suppress concentration‐caused emission quenching and exciton annihilation. In addition, abundant C—H···π and C—H···O hydrogen bonds can be observed in crystal, but no obvious π–π interaction is found (Figure 1c). The cooperative effect of these weak hydrogen bonds can rigidify molecular conformation and restrict intramolecular motion, in favor of reducing the motional nonradiative transitions and increasing the PL quantum yields (*Φ*_PL_) in the solid state.^19^, ^20^

### 2.3 Photophysical Properties

**Figure 2:**
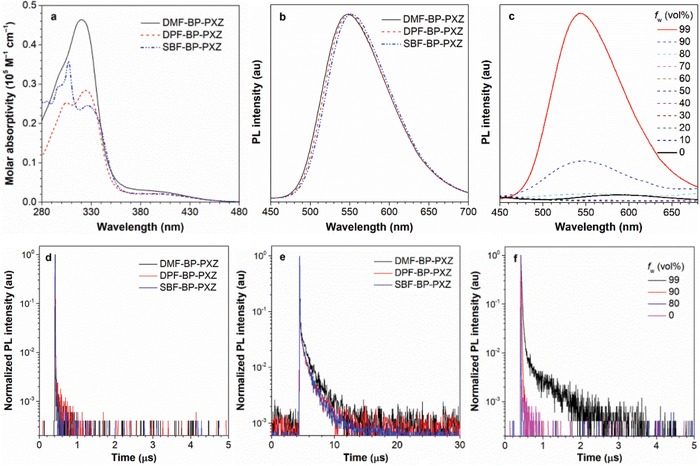
a) Absorption spectra in THF solutions (10^−5^ m), b) PL spectra in neat films of DMF‐BP‐PXZ, DPF‐BP‐PXZ, and SBF‐BP‐PXZ. Transient PL decay spectra of these molecules in d) THF solutions (10^−5^ m) and e) neat films, measured at 300 K under nitrogen. c) PL spectra and f) transient PL decay spectra of DMF‐BP‐PXZ in THF/water mixtures with different water fractions (*f*_w_), measured under nitrogen.

As depicted in **Figure** **2**a, the absorption spectra of DMF‐BP‐PXZ, DPF‐BP‐PXZ, and SBF‐BP‐PXZ in dilute THF solutions show main peaks at ≈324 nm, associated to the π–π* transition of the molecules. Weak and broad absorption tails in the range of 380–460 nm are observed, which are assigned to the charge transfer (CT) from the peripheral donor unit to the central benzoyl acceptor. The PL peaks of these molecules in THF solutions are found at ≈595 nm with *Φ*_PL_ values as low as 1.8–2.8%. And the transient PL decay spectra reveal that they only possess prompt components with short lifetimes of 1.8–3.2 ns in normal fluorescence scale (Figure 2d). Interestingly, along with the aggregate formation upon addition of a large volume of water into the THF solutions, the PL intensities of these molecules are enhanced and the fluorescence lifetimes become much longer with notable delayed fluorescence (Figure 2c,f and Figures S3 and S4, Supporting Information). The increased ratio and lifetime of delayed fluorescence as the aggregates form clearly manifest the prominent AIDF property (Table S1, Supporting Information).

To factually assess the performance of these molecules, their photophysical properties were further investigated in neat films as well as doped films (**Table** **1**). At room temperature, the emission bands of their neat films locate in the yellow region with PL peaks centered at ≈550 nm (Figure 2b). The *Φ*_PL_ values of these neat films are greatly increased to 45.4–48.7%, much higher than those in solutions, which can be attributed to the suppression of intramolecular motions and thus the block of nonradiative decay channel. The fact that the delayed components of these molecules are indiscernible in solutions but the delayed fluorescence property becomes more distinct with long lifetimes of 1.1–1.4 μs in vacuum‐deposited neat films (Figure 2d) further validates their AIDF nature.

**Table 1:** Photophysical properties of DMF‐BP‐PXZ, DPF‐BP‐PXZ, and SBF‐BP‐PXZ

	Solna)				Neat filmb)				Doped filmb)			
	λ_abs_ [nm]	λ_em_ [nm]	Φ_PL_ c) [%]	τd) [ns]	λ_em_ [nm]	Φ_PL_ c) [%]	τ_prompt_ e) [ns]	τ_delayed_ e) [µs]	λ_em_ [nm]	Φ_PL_ c) [%]	τ_prompt_ e) [ns]	τ_delayed_ e) [µs]
DMF‐BP‐PXZ	406, 320	595	2.6	2.3	548	45.4	30.2	1.4	534	73.4	31.0	2.1
DPF‐BP‐PXZ	408, 324	596	2.8	3.2	550	48.7	27.9	1.1	536	69.5	29.3	2.3
SBF‐BP‐PXZ	407, 327	596	1.8	1.8	551	45.5	27.5	1.1	542	73.0	30.5	2.1

^a)^ Measured in THF solution (10^−5^ m) at room temperature

^b)^ Deposited on a quartz substrate

^c)^ Absolute PL quantum yield determined by a calibrated integrating sphere under nitrogen at room temperature

^d)^ Mean fluorescence lifetime evaluated at 300 K under nitrogen

^e)^ Fluorescence lifetimes of prompt (τ_prompt_) and delayed (τ_delayed_) decay components evaluated at 300 K under nitrogen.

To gain deeper insights into the mechanism of the interesting AIDF behavior, we further compare the photophysical transition rates of DMF‐BP‐PXZ, DPF‐BP‐PXZ, and SBF‐BP‐PXZ in solutions and in neat films. The radiative decay rate (*k*_F_), internal conversion rate (*k*_IC_), intersystem crossing rate (*k*_ISC_), and reverse intersystem crossing rate (*k*_RISC_) were calculated by the equations showed in Supporting Information.^21^, ^22^, ^23^ As listed in **Table** **2**, these molecules have close *k*_F_ values in solutions and in neat films, for the molecular conjugation is barely changed in both states. However, the *k*_IC_ values are decreased from solutions (3.1 × 10^8^ – 5.5 × 10^8^ s^−1^) to neat films (1.3 × 10^7^ – 1.6 × 10^7^ s^−1^), and become comparable to the *k*_ISC_ values (7.7 × 10^6^ – 9.7 × 10^6^ s^−1^). The intramolecular rotations are highly active in solutions, which can act as the major IC channels to rapidly and efficiently deactivate the excited state via nonradiative relaxation. In that case, the IC process is overwhelming, and, undoubtedly, the ISC and reverse intersystem crossing (RISC) processes can hardly occur, accounting for the disappearance of delayed fluorescence. However, in the aggregated state, the intramolecular motions are restricted greatly, and thus the nonradiative IC channels are blocked as evidenced by the reduced *k*_IC_ values. In consequence, ISC and RISC processes are more ready to occur under the premise of the small singlet–triplet energy splitting, allowing the prominent delayed fluorescence.

**Table 2:** Photophysical parameters of DMF‐BP‐PXZ, DPF‐BP‐PXZ, and SBF‐BP‐PXZ in solutions and neat films

	DMF‐BP‐PXZ		DPF‐BP‐PXZ		SBF‐BP‐PXZ	
	Soln	Film	Soln	Film	Soln	Film
*Φ*_prompt_[%]a)	2.6	32.1	2.8	37.5	1.8	35.9
*Φ*_delayed_[%]	–	13.3	–	11.2	–	9.6
*Φ*_ISC_ [%]	–	29.3	–	23	–	21.1
*Φ*_RISC_ [%]	–	45.4	–	48.7	–	45.5
*k*_F_ [× 10^6^ s^−1^]	11.1	10.6	8.8	13.5	10.1	13
*k*_IC_ [× 10^7^ s^−1^]	41.7	1.3	30.5	1.4	55.2	1.6
*k*_ISC_ [× 10^6^ s^−1^]	–	9.7	–	8.2	–	7.7
*k*_RISC_[× 10^6^ s^−1^]	–	1.0	–	1.2	–	1.1

^a)^ Abbreviations: *Φ*_prompt_ and *Φ*_delayed_ = prompt and delayed components, respectively, determined from the total *Φ*_PL_ and the proportion of the integrated area of each component in the transient spectra to the total integrated area; *Φ*_ISC_ = the intersystem crossing quantum yield; *k*_F_ = fluorescence decay rate; *k*_IC_ = internal conversion rate; *k*_ISC_ = intersystem crossing rate; *k*_RISC_ = reverse intersystem crossing rate.

### 2.4 Nanosecond Transient Absorption Spectroscopy

**Figure 3:**
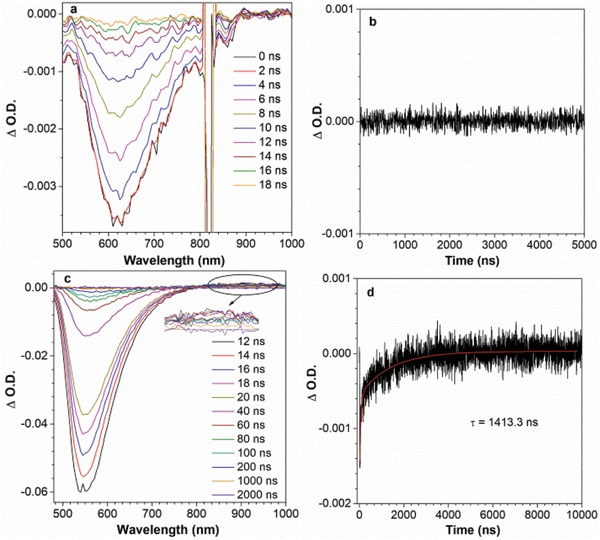
Nanosecond TA spectra of DMF‐BP‐PXZ in a) degassed THF solution (10^−5^ m) and c) the vacuum‐deposited neat film. Decay curves of DMF‐BP‐PXZ in b) degassed THF solution (10^−5^ m) and d) the vacuum‐deposited neat film at 940 nm (λ_ex_ = 410 nm).

To validate the above hypothesis, we conducted the nanosecond transient absorption (TA) spectral measurements for DMF‐BP‐PXZ in deaerated THF solution and vacuum‐deposited neat film (**Figure** **3**). Upon pulsed laser excitation, a dominant bleaching band at 545 nm appears in neat film, which is assigned to the stimulated emission. Besides, a broad excited‐state absorption (ESA) band in the range of 800–1000 nm can be observed in neat film, indicative of the formation of triplet‐excited state. The lifetime of this triplet state is determined to be 1413.3 ns, obtained by fitting the time trace of the ESA band at 940 nm, which is close to the lifetime of delayed fluorescence of DMF‐BP‐PXZ in neat film (1.4 µs). This clearly evidences the occurrence of ISC process. In deaerated THF solution, the bleaching band from the stimulated emission is recorded at around 620 nm, but the transient spectra show no signal indicating the formation of triplet state. This reveals that DMF‐BP‐PXZ hardly experiences ISC process in solution because of the much faster IC process. According to the results of the nanosecond TA experiments, the hypothetical mechanism of AIDF phenomenon has been further confirmed.

### 2.5 Anti‐Kasha Behavior and Excited State Dynamics

Density functional theory and time‐dependent density functional theory simulations were employed to deeply depict the underlying mechanism of the emission property. Meanwhile, the combined quantum mechanics and molecular mechanics (QM/MM) method with two‐layer ONIOM approach was used to simulate the photophysical properties in the solid state, where the central molecule is defined as the active QM part, and the surrounding ones are all rigid as the MM part.^24^, ^25^, ^26^ The computational model was constructed based on the X‐ray crystal structure of the representative molecule DMF‐BP‐PXZ. As the excited state properties of D‐A type molecules are strongly dependent on the functional, it is vital to select an appropriate functional. Initially, the geometric structures of DMF‐BP‐PXZ for the ground state (S_0_) and the lowest singlet excited state (S_1_) are both theoretically optimized in solid phase by BMK functional with 6‐31G(d) basis set. On the basis of the optimal structure, the oscillator strength (*f*)^27^ for the electronic relaxation from S_1_ is calculated reliably. A particularly surprising result is that the oscillator strength of S_1_ is almost zero (*f* = 0.0006), indicating S_1_ is nonemissive, which is opposite to the common belief and most reported experimental findings. Then we tried various functionals including M06, M062X, MPW1B95, PBE33, PBE38, and PBE0 that have different Hartree–Fock hybrid proportions to optimize the structure, and the *f* values are still nearly equal to zero for the electronic transition from S_1_ to S_0_ (Table S2, Supporting Information), disclosing that S_1_ is indeed a dark state. Instead, the efficient emission of DMF‐BP‐PXZ actually should originate from the radiative relaxation of the higher energy electronic excited state, which doesn't obey Kasha's rule.^28^, ^29^, ^30^, ^31^, ^32^ Next, the second excited state (S_2_) geometry was further optimized using the MPW1B95 and PBE0 functional. It turns out that the S_2_ has a much larger oscillator strength than S_1_ (Table S3, Supporting Information). Furthermore, the emission wavelength of DMF‐BP‐PXZ in solid calculated with the PBE0 functional is 525 nm with a relatively large *f* value of 0.0523, which is in better agreement with the experimental result (525 nm in crystal). These findings demonstrate the emission of DMF‐BP‐PXZ is closely associated to the radiative decay of S_2_ indeed. In other words, DMF‐BP‐PXZ has anti‐Kasha behavior. This unusual anti‐Kasha emission may be caused by the competition between electron‐donating PXZ and 9,9‐dimethylfluorene in forming excited CT state as well as excited state geometry with the electron‐withdrawing benzoyl core.

**Figure 4:**
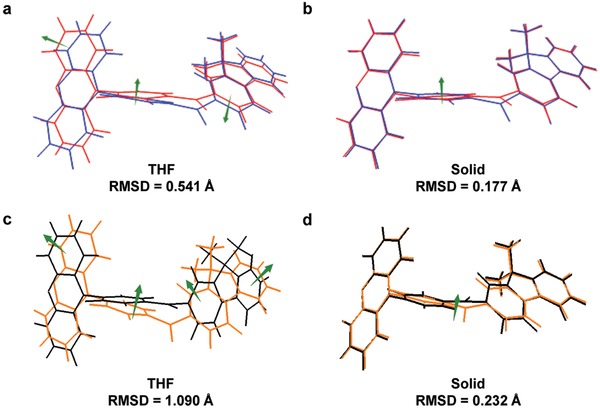
Geometry comparisons between S_0_ (blue) and S_1_ (red) in a) THF and b) solid phase; geometry comparisons between S_2_ (black) and T_3_ (orange) in c) THF and d) solid phase.

As mentioned above, DMF‐BP‐PXZ exhibits single‐exponential decay profiles with sole prompt fluorescence in THF solution, but the delayed fluorescence becomes more obvious as the aggregates form. The calculated results indicate that the S_1_ is a dark state, whereas the S_2_ is an emissive one. So, the radiative decay of S_2_ is closely relevant to the strong emissions of these molecules in solid. The calculated radiative rate (*k*_F_) from S_2_ to S_0_ in solid phase is 1.3 × 10^7^ s^−1^, in good agreement with the experimental value of 1.1 × 10^7^ s^−1^. To better understand this photophysical phenomenon, the nonradiative decay process in solution and in solid are further considered. Therefore, the rates of IC processes in both states have to be calculated because the IC process is mainly responsible for the nonradiative decay of the excited state. According to the energy‐gap law, an excited molecule at S_2_ undergoes IC process to S_1_ and then to S_0_, while the IC process from S_2_ to S_1_ is much faster than that from S_1_ to S_0_, which means the entire rate of nonradiative decay process of S_2_ → S_1_ → S_0_ is virtually determined by the rate of S_1_ → S_0_.^33^ Since it is difficult for us to obtain the exact value of *k*_IC_ of S_2_ → S_1_ by calculation, we just use the *k*_IC_ of S_1_ → S_0_ to replace that of S_2_ → S_1_ → S_0_ for the discussion, which is reasonable. As predicted, the *k*_IC_ of S_1_ → S_0_ in solid (8.8 × 10^6^ s^−1^) is about 4 orders of magnitude smaller than that in solution (3.1 × 10^10^ s^−1^), manifesting that the IC process is responsible for the weak emission in solution indeed. This should be owing to the restricted geometry changes in solid, and the intuitive comparisons of S_0_ and S_1_ geometries also clearly illustrate the degree of deformation (**Figures** **4**a,b). The root of the mean of squared displacement (RMSD) between two states is also calculated using Multiwfn to quantitatively characterize the geometric change.^34^ Obviously, the reduced structural deformation with smaller RMSD values are found in solid than in solution, owing to the molecular structural rigidification and intramolecular motion restriction by physical constraint in solid.

By further calculating the *k*_ISC_ values, it is found that the recombination energy versus the normal‐mode frequency between S_2_ and the proximal triplet states are too large to be applicable for the molecular materials property prediction package (MOMAP) program in THF. Instead, the *k*_ISC_ values between an initial singlet state I and a final triplet state F can also be described in the well‐known Fermi Golden rule expression^35^, ^36^, ^37^:

(1) $$k_{\mathrm{ISC}}=\frac{{〈}^{1}\psi_{I}^{0}|H_{\mathrm{SO}}{|{}^{3}\psi_{F}^{0}〉}^{2}}{\hbar }\sqrt{\frac{\pi }{k_{B}T\lambda }}\exp\left[-\frac{{\left(\lambda +\Delta E\right)}^{2}}{4\lambda k_{B}T}\right]$$

where *k*_B_ is Boltzmann constant and *T* is temperature, which is set to 300 K. ${〈}^{1}\psi_{I}^{0}|H_{\mathrm{SO}}{|}^{3}\psi_{F}^{0}〉$ is the expectation value of spin orbit coupling (SOC) and λ is the Marcus reorganization energy. ∆*E* is the energy difference between the initial and final states.

**Figure 5:**
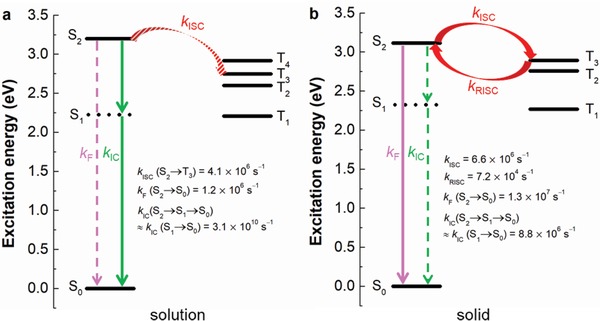
Adiabatic excitation energies for DMF‐BP‐PXZ a) in THF solution and b) in solid.

Fermi Golden rule expression is the simplest and the most commonly employed intersystem crossing rate formalism. Accordingly, the SOC, ∆*E*, and reorganization energy values have significant impacts on the *k*_ISC_ values. For DMF‐BP‐PXZ, the primary parameters are summarized in Tables S4 and S5 in the Supporting Information, and the adiabatic excitation energies in solution and in solid are shown in **Figure** **5**. It can be seen that S_2_→T_3_ transition is dominant according to the calculated *k*_ISC_ values. And the theoretical result in solid (6.6 × 10^6^ s^−1^) is on the same order as the experimental value (9.7 × 10^6^ s^−1^), clearly indicating the validity of this method in evaluating the exciton transformation channels for ISC process. It is worth noting that the *k*_ISC_ value in solid is slightly higher than that in solution, which can be attributed to the stronger SOC (0.948 cm^−1^) and smaller reorganization energy (0.76 eV) in solid. Therein, reorganization energy plays a more crucial role in ISC process, and the smaller reorganization energy in solid is consistent with the greatly reduced geometric changes between S_2_ and T_3_ in solid relative to those in solution (Figures 4c,d).

**Figure 6:**
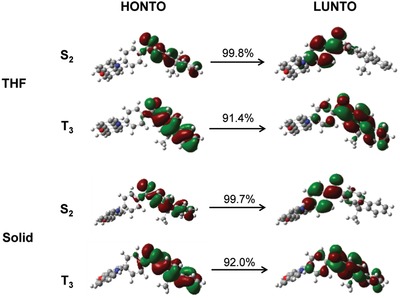
Transition characteristics for S_2_ and T_3_ sates of DMF‐BP‐PXZ in THF solution and in solid phase. The value above every arrow represents the ratio of depicted NTOs in corresponding transition.

Additionally, the transition character of excited states plays an important role in determining the excited state properties. To provide a precise picture of the excited states, natural transition orbitals (NTOs) analysis are carried out to give a compact orbital representation based on the singular value decomposition of the one‐particle transition density matrix. The highest occupied natural transition orbital (HONTO) and the lowest unoccupied natural transition orbital (LUNTO) of DMF‐BP‐PXZ at S_2_ and T_3_ are investigated in THF and in solid (**Figure** **6**). As for S_2_, it is a typical CT state which is very sensitive to the polarity of the environment, but not local excitation (LE) state.^38^ The HONTO is localized in 9,9‐dimethylfluorene unit and LUNTO is mainly distributed on the benzoyl unit. A significant LE feature can be found for T_3_. Recent theoretical and spectroscopic studies by several groups have revealed that the delayed fluorescence efficiency can be effectively mediated by mixing the ^1^CT state with the energetically close‐lying ^3^LE state.^39^, ^40^, ^41^, ^42^ Through the theoretical calculations, the energy gap between S_2_ and T_3_ is so small (0.22 eV) in solid that RISC process becomes highly favorable in energy for exciton transformation according to the energy gap law.^43^ Consistently, the *k*_RISC_ from T_3_ to S_2_ is evaluated to be 7.2 × 10^4^ s^−1^ (Table S5, Supporting Information), namely, T_3_ can be converted to S_2_ efficiently in solid, perfectly agreeing with the experimental findings, and also supporting the anti‐Kasha emission.

Crucially, these computational results can well explain the experimental observations, and reveal the dynamical mechanism of AIDF phenomenon. The transition character of the excited state can be significantly influenced by the environment of the fluorogenic molecules. From THF solution to the solid state, the nonradiative relaxation of the excited states via IC channel is greatly suppressed and the ISC process is promoted simultaneously, which is beneficial to generate delayed fluorescence under the premise of small singlet−triplet energy splitting, that is the restriction of intramolecular motion and molecular structural rigidification contribute significantly to the delayed fluorescence.

### 2.6 Electroluminescence

The interesting AIDF property of these materials inspired us to investigate their EL performance in OLEDs. Three‐layer nondoped devices (Device I) were fabricated with the configuration of indium tin oxide (ITO)/TAPC (25 nm)/emitter (35 nm)/TmPyPB (55 nm)/LiF (1 nm)/Al, where neat films of these materials are adopted as the emitting layers. 1,1′‐Bis(di‐4‐tolylaminophenyl)cyclohexane (TAPC) and 1,3,5‐tri(*m*‐pyrid‐3‐yl‐phenyl)benzene (TmPyPB) functioned as hole‐ and electron‐transporting layers, respectively. The EL spectra, current density–voltage–luminance (J–V–L) characteristics, and current efficiency versus luminance plots of the nondoped OLEDs are depicted in Figure S7 in the Supporting Information, and the key values of EL performances are listed in **Table** **3**. The nondoped OLEDs based on these AIDF materials are turned on at low voltages of 2.5–2.7 V and display strong yellow EL emissions peaking at around 560 nm, suggesting efficient carrier injection and transport in such a simple device configuration. The EL spectra are stable at different current densities (Figure S8, Supporting Information). Remarkably, the high maximum external quantum (η_ext_), current (η_c_), and power efficiencies (η_p_) of up to 14.3%, 41.6 cd A^−1^, and 45.0 lm W^−1^ are obtained in the device of DPF‐BP‐PXZ. More importantly, the device still retains excellent η_ext_ and η_c_ of 14.1% and 41.4 cd A^−1^, respectively, at 1000 cd m^−2^. The corresponding roll‐off of external quantum yield is as small as 1.4%, indicative of the greatly advanced efficiency stability. Owing to the highly twisted molecular structures and fast RISC process, the short‐range Dexter energy transfer quenching of high‐concentration excitons can be diminished greatly because of the relatively long distance between excitons that are located at the center of branched AIDF luminogens (benzoyl group). And the triplet excitons can be converted to singlet excitons efficiently, followed by radiative decay for light emission. In consequence, the annihilation of triplet excitons is well reduced. On this account, the nondoped OLEDs using DMF‐BP‐PXZ or SBF‐BP‐PXZ as emitting layers also exhibit good efficiency stability with small efficiency roll‐off of 6.0% and 0.8%, respectively, at 1000 cd m^−2^ luminance. Besides, the single‐carrier devices of these AIDF emitters have been fabricated (Figure S9, Supporting Information). The results indeed evidence that these materials, especially DMF‐BP‐PXZ and DPF‐BP‐PXZ, possess comparable electron‐ and hole‐transporting abilities, which is conducive to achieving balanced holes and electrons in OLEDs, and thus realizing high EL efficiencies.

**Table 3:** EL performances of OLEDs based on DMF‐BP‐PXZ, DPF‐BP‐PXZ, and SBF‐BP‐PXZ

Emitter	Maximum valuesa)					Values at 1000 cd m^−2^					
	*V*_on_ [V]	η_c_ [cd A^−1^]	η_P_ [lm W^−1^]	η_ext_ [%]	*L* [cd m^−2^]	*V* [V]	η_C_ [cd A^−1^]	η_P_ [lm W^−1^)	η_ext_ [%]	RO [%]	CIE [*x*,*y*]
Nondoped device I											
DMF‐BP‐PXZ	2.7	39.9	38.0	13.3	27 331	4.9	37.4	24.0	12.5	6.0	(0.440, 0.543)
DPF‐BP‐PXZ	2.6	41.6	45.0	14.3	31 422	5.0	41.4	26.0	14.1	1.4	(0.458, 0.530)
SBF‐BP‐PXZ	2.5	36.8	37.9	12.3	33 990	4.0	36.7	28.8	12.2	0.8	(0.456, 0.528)
Doped device II											
DMF‐BP‐PXZ	2.7	60.6	55.6	18.6	53 013	4.6	58.8	42.4	18.2	2.2	(0.404, 0.565)
DPF‐BP‐PXZ	2.7	62.3	59.9	19.0	77 480	4.4	61.5	43.9	18.8	1.1	(0.399, 0.564)
SBF‐BP‐PXZ	2.7	62.3	62.9	19.4	113 145	4.2	61.8	46.2	19.2	1.0	(0.419, 0.557)

^a)^ Abbreviations: *V*_on_ = turn‐on voltage at 1 cd m^−2^; η_c_ = current efficiency; η_p_ = power efficiency; η_ext_ = external quantum efficiency; *L* = luminance; RO = external quantum efficiency roll‐off from maximum value to that at 1000 cd m^−2^; CIE = Commission Internationale de I'Eclairage coordinates. Device I (nondoped OLEDs): ITO/TAPC (25 nm)/DMF‐BP‐PXZ or DPF‐BP‐PXZ or SBF‐BP‐PXZ (35 nm)/TmPyPB (55 nm)/LiF (1 nm)/Al; Device II (doped OLEDs): ITO/TAPC (25 nm)/30 wt% DMF‐BP‐PXZ or 30 wt% DPF‐BP‐PXZ or 30 wt% SBF‐BP‐PXZ : CBP (35 nm)/TmPyPB (55 nm)/LiF (1 nm)/Al.

Furthermore, we also fabricated doped OLEDs (Device II) employing doped films of 30 wt% DMF‐BP‐PXZ, DPF‐BP‐PXZ, or SBF‐BP‐PXZ in 4,4′‐bis(carbazol‐9‐yl)biphenyl (CBP) host as the light‐emitting layers with the same configuration of ITO/TAPC (25 nm)/emitter (35 nm)/TmPyPB (55 nm)/LiF (1 nm)/Al. These doped OLEDs with a high doping concentration also show excellent EL performances with a low turn‐on voltage of 2.7 V and a highest luminance of 113 145 cd m^−2^. The high maximum η_ext_ of 18.6–19.4%, η_c_ of 60.6–62.3 cd A^−1^, and η_p_ of 55.6–62.9 lm W^–1^ are attained (Table 3 and Figure S10, Supporting Information). And they are still capable of retaining very high EL efficiencies at 1000 cd m^−2^ with negligible efficiency roll‐off, which are much superior to most doped OLEDs using TADF emitters.

Under electrical excitation, the triplet excitons in OLEDs based on AIDF emitters are directly generated by carrier recombination and are subsequently up‐converted to the radiative singlet excitons via an efficient RISC process. Consequently, the theoretical maximum η_ext_ values can be deduced by the following equations^44^:

(2) $$\eta_{\mathrm{ext}}=\eta_{\mathrm{int}}\times \eta_{\mathrm{out}}$$

(3) $$\eta_{\mathrm{int}}=\gamma \times \left[\eta_{S}\times \phi_{\mathrm{prompt}}+\left(\eta_{S}\times \phi_{\mathrm{ISC}}+\eta_{T}\right)\times \phi_{\mathrm{RISC}}\right]$$

where η_int_ denotes the internal quantum efficiency, η_out_ is the optical out‐coupling factor (typically 0.2–0.3), γ is the charge balance factor (ideally γ = 1.0), and η_S_ and η_T_ are the fractions of singlet and triplet excitons (25% and 75%, respectively). By assuming the optical out‐coupling efficiency of 0.3, the corresponding theoretical maximum η_ext_ values are estimated to be 13.6%, 14.6%, and 13.7% for the nondoped OLEDs of DMF‐BP‐PXZ, DPF‐BP‐PXZ, and SBF‐BP‐PXZ, respectively. These theoretical values agree well with the experimentally obtained η_ext_ values (Table 3), revealing that the carrier is well balanced and high exciton utilization is indeed achieved in the AIDF emitters.

## 3 Conclusion

In summary, we have developed a series of new AIDF materials by combining an electron‐withdrawing benzoyl core and the electron‐donating PXZ and fluorene derivatives. These AIDF materials can be readily synthesized with high yields so that they are applicable for the large‐scale commercial applications. They exhibit weak emissions without delayed fluorescence in solutions but strong emissions with obvious delayed components upon aggregate formation or in neat films, exhibiting the typical AIDF characteristics. The photophysical measurements and theoretical calculations demonstrate that this phenomenon can be ascribed to the suppression of IC channels and the promotion of ISC process in solid, which essentially benefits from restriction of intramolecular motion and rigidification of molecular structure. Meanwhile, the theoretical calculations reveal that the S_1_ of DMF‐BP‐PXZ is a dark state and its efficient emission emanates from the higher energy electronic excited states (e.g., S_2_), demonstrating an anti‐Kasha behavior. Moreover, the ISC and RISC processes can occur efficiently between S_2_ and T_3_ in solid, and the radiative decay of S_2_ furnishes prompt and delayed fluorescence. Owing to the AIDF property, these materials can achieve high exciton utilization and greatly depress exciton annihilation in both nondoped and doped OLEDs, rendering excellent EL efficiencies and extremely small efficiency roll‐off. These important insights gained in this work will shed light on the design principles for efficient light emitters for OLEDs and also open a new avenue to the utilization of higher energy electronic excited states for light emission.

## Conflict of Interest

The authors declare no conflict of interest.

## Supporting information

Supplementary
