# Supplementary material for: Mechanical Insights into Aggregation‐Induced Delayed Fluorescence Materials with Anti‐Kasha Behavior
Source: Adv Sci (Weinh). 2018 Dec 7;6(3):1801629. doi: 10.1002/advs.201801629 (PMC6364497; doi:10.1002/advs.201801629)
Supplement: Supplementary file 1 — Supplementary [file ADVS-6-1801629-s001.pdf]

Copyright WILEY-VCH Verlag GmbH & Co. KGaA, 69469 Weinheim, Germany, 2018.

ADVANCED SCIENCE
Open Access

Supporting Information

for *Adv. Sci.,* DOI: 10.1002/advs.201801629

Mechanical Insights into Aggregation-Induced Delayed Fluorescence Materials with Anti-Kasha Behavior

Jingjing Guo, Jianzhong Fan, Lili Lin, Jiajie Zeng, Hao Liu, Chuan-Kui Wang, Zujin Zhao, and Ben Zhong Tang**

Copyright WILEY-VCH Verlag GmbH & Co. KGaA, 69469 Weinheim, Germany, 2016.

Supporting Information

Mechanical Insights into Aggregation-Induced Delayed Fluorescence Materials with Anti-Kasha Behavior

Jingjing Guo,‡ Jianzhong Fan,‡ Lili Lin, Jiajie Zeng, Hao Liu, Chuan-Kui Wang, Zujin Zhao, and Ben Zhong Tang**

1. General information

All the chemicals and reagents were purchased from commercial sources and used as received without further purification. The final products were subjected to vacuum sublimation to further improve purity before photoluminescence (PL) and electroluminescnce (EL) properties investigations. ^{1}H and ^{13}C NMR spectra were measured on a Bruker AV 500 spectrometer in $CDCl_3$ or DMSO-d_6 at room temperature. High resolution mass spectra (HRMS) were recorded on a GCT premier CAB048 mass spectrometer operating in MALDI-TOF mode. Single crystal X-ray diffraction intensity data were collected at 173 K on a Bruker–Nonices Smart Apex CCD diffractometer with graphite monochromated MoKα radiation. Processing of the intensity data was carried out using the SAINT and SADABS routines, and the structure and refinement were conducted using the SHELTL suite of X-ray programs (version 6.10). UV-vis absorption spectrum was measured on a Shimadzu UV-2600 spectrophotometer. PL spectra were recorded on a Horiba Fluoromax-4 spectrofluorometer. PL quantum yields were measured using a Hamamatsu absolute PL quantum yield spectrometer C11347 Quantaurus_QY. Transient PL decay spectra were measured using Quantaurus-Tau fluorescence lifetime measurement system (C11367-03, Hamamatsu Photonics Co., Japan). Cyclic voltammogram was measured in a solution of tetra-*n*-butylammonium hexafluorophosphate (Bu_4NPF_6, 0.1 M) in acetonitrile containing the sample

at a scan rate of 100 mV s^{-1}. Three-electrode system (Ag/Ag^+, platinum wire and glassy carbon electrode as reference, counter and work electrode respectively) was used in the CV method. HOMO = −[E_{ox} + 4.8] eV, and LUMO = −[E_{re} + 4.8] eV. E_{ox} and E_{re} represent the onset oxidation and reduction potentials relative to Fc/Fc^+, respectively.

The ground-state geometries were optimized by density functional theory (DFT) method and the photophysical properties of excited states were studied by time-dependent density functional theory (TDDFT). The combined quantum mechanics and molecular mechanics (QM/MM) method with two-layer ONIOM approach was used to simulate the properties in the solid state. The PBE0 functional was adopted in the calculations of DMF-BP-PXZ molecule. Based on all the geometric and electronic structure information obtained above, the radiative decay rate was calculated by Einstein spontaneous emission equation, which is written as

$$k_r = \frac{f \Delta E_{fi}^2}{1.499} \quad (1)$$

Here, f is oscillator strength and ΔE_{fi} is the vertical emission energy with the unit of wavenumber (cm^{-1}).

As for the non-radiative decay rate k_{nr}, it can be expressed as the following form according to the Fermi's golden rule (FGR) and first-order perturbation theory.

$$k_{nr} = \frac{2\pi}{\hbar}\sum_{kl} R_{kl} Z_i^{-1} \sum_{vu} e^{-\beta E_{iv}} \langle \Theta_{fu} | \hat{P}_{fk} | \Theta_{iv} \rangle \langle \Theta_{iv} | \hat{P}_{fl} | \Theta_{fu} \rangle \delta(E_{iv} - E_{fu}) \quad (2)$$

Here, $R_{kl} = \langle \Phi_f | \hat{P}_{fk} | \Phi_i \rangle \langle \Phi_i | \hat{P}_{fl} | \Phi_f \rangle$ is the non-adiabatic electronic coupling. Z_i is the partition function and $\hat{P}_{fk} = -i\hbar \frac{\partial}{\partial Q_{fk}}$ represents the normal momentum operator of the kth normal mode in the final electronic state. Φ and Θ are the electronic state and nuclear vibrational wave function respectively. u and v are vibrational quantum numbers. f and i represent the final and initial state respectively. Furthermore, applying the Fourier transform of the delta function and Franck-Condon principle, the equation 2 can be written as

$$k_{nr} = \sum_{kl} \frac{1}{\hbar^2} R_{kl} \int_{-\infty}^{\infty} dt \left[e^{i\omega_{if}t} Z_i^{-1} \rho_{ic,kl}(t,T) \right] \quad (3)$$

Where $\rho_{ic,kl}(t,T)$ is the thermal vibration correlation function (TVCF) and it can be expressed as $\rho_{ic,kl}(t,T) = Tr(\hat{P}_{fk} e^{-i\tau_f \hat{H}_f} \hat{P}_{fl} e^{-i\tau_i \hat{H}_i})$. All these parameters can be calculated by MOMAP (Molecular Materials Property Prediction Package).

Besides, the intersystem crossing (ISC) and reverse intersystem crossing (RISC) rate constants were calculated in the Marcus expression.

2. Synthesis and characterization

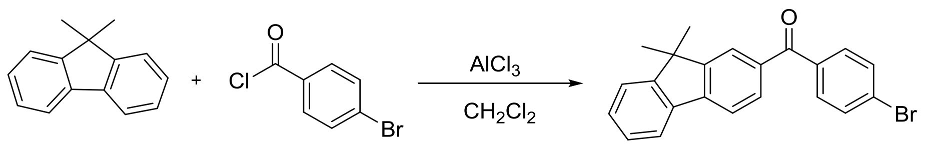

DMF-BP-Br

(4-Bromophenyl)(9,9-dimethylfluoren-2-yl)methanone (DMF-BP-Br): 4-Bromobenzoyl chloride (0.78 g, 3.6 mmol), 9,9-dimethyl-9H-fluorene (0.58 g, 3 mmol) and 25 mL dry dichloromethane were mixed together under stirring at ambient conditions. Then anhydrous $AlCl_3$ (0.48 g, 3.6 mmol) was added into the mixture slowly and followed by stirring for 3 h at 40 °C. Then, the reaction was quenched by poured into 200 mL ice-HCl solution, and the aqueous layer was separated from the organic layer. Then the aquatic phase was further extracted with dichloromethane for several times. The combined organic layers were washed twice with water (extracted the aquatic phases again if needed), and then dried over anhydrous Na_2SO_4. After filtration and solvent evaporation under reduced pressure, the residue was purified by silica-gel column chromatography using dichloromethane/petroleum as eluent. DMF-BP-Br was obtained as a white solid in 87% yield. ^{1}H NMR (500 MHz, $CDCl_3$, δ): 7.91 (d, J = 1.0 Hz, 1H), 7.82–7.68 (m, 5H), 7.67–7.63 (m, 2H), 7.50–7.46 (m, 1H), 7.43–7.36 (m, 2H), 1.52 (s, 6H). ^{13}C NMR (125 MHz, $CDCl_3$, δ): 195.63, 154.78, 153.83, 143.98, 137.83, 136.99, 135.82, 131.58, 131.51, 130.04, 128.70, 127.31, 127.18, 124.29, 122.88, 121.02,

119.57, 47.08, 26.96. HRMS (MALDI-TOF): m/z [M$^+$] calcd. $C_{22}H_{17}BrO$, 376.0463; found, 376.0608.

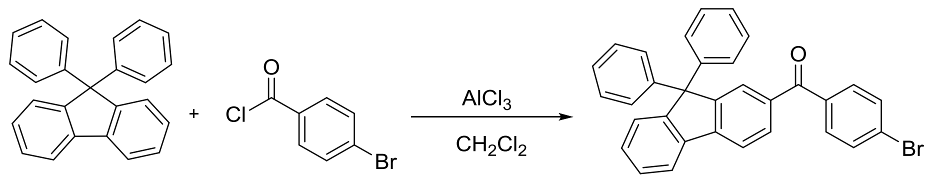

DPF-BP-Br

(4-Bromophenyl)(9,9-diphenylfluoren-2-yl)methanone (DPF-BP-Br): The procedure was analogous to that described for DMF-BP-Br. White solid, yield 91%. ^{1}H NMR (400 MHz, DMSO-d_6, δ): 8.15–8.05 (m, 2H), 7.81–7.72 (m, 4H), 7.66–7.61 (m, 2H), 7.56–7.40 (m, 3H), 7.34–7.22 (m, 6H), 7.17–7.09 (m, 4H). ^{13}C NMR (125 MHz, $CDCl_3$, δ): 195.05, 152.20, 151.43, 145.14, 144.79, 138.78, 136.56, 136.10, 131.59, 131.48, 130.17, 129.12, 128.39, 128.17, 128.03, 127.86, 127.30, 126.97, 126.51, 121.21, 120.03, 65.57. HRMS (MALDI-TOF): m/z [M$^+$] calcd. $C_{32}H_{21}BrO$, 500.0776; found, 500.1129.

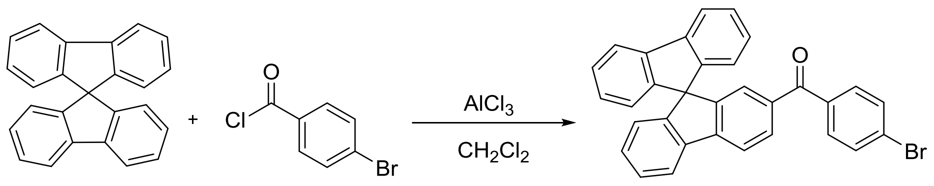

SBF-BP-Br

9,9'-Spirobi[fluoren]-2-yl(4-bromophenyl)methanone (SBF-BP-Br): The procedure was analogous to that described for DMF-BP-Br. White solid, yield 84%. ^{1}H NMR (500 MHz, $CDCl_3$, δ): 7.94–7.89 (m, 2H), 7.84 (d, J = 7.6 Hz, 2H), 7.78 (dd, J = 8.0, 1.6 Hz, 1H), 7.51 (s, 4H), 7.43–7.35 (m, 3H), 7.22–7.16 (m, 2H), 7.14–7.09 (m, 2H), 6.78 (d, J = 7.6 Hz, 1H), 6.73 (d, J = 7.6 Hz, 2H). ^{13}C NMR (125 MHz, $CDCl_3$, δ): 194.99, 150.05, 149.25, 147.70, 146.34, 141.87, 140.30, 136.57, 136.32, 131.44, 131.39, 130.67, 129.26, 128.03, 127.91, 127.17, 125.70, 124.32, 123.88, 120.96, 120.27, 119.64, 65.90. HRMS (MALDI-TOF): m/z [M$^+$] calcd. $C_{32}H_{19}BrO$, 498.0619; found, 498.0593.

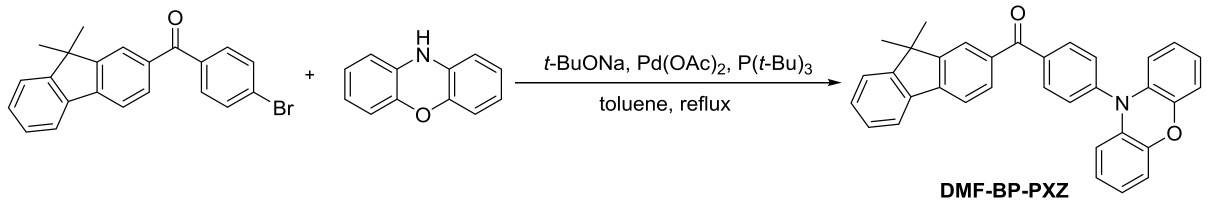

DMF-BP-PXZ

(4-(Phenoxazin-10-yl)phenyl)(9,9-dimethylfluoren-2-yl)methanone (DMF-BP-PXZ): A mixture of DMF-BP-Br (0.75 g, 2mmol), phenoxazine (0.55 g, 3 mmol), t-BuONa (0.58 g, 6 mmol), $P(t\text{-}Bu)_3$ (0.37 ml, 0.16 mmol), $Pd(OAc)_2$ (36 mg, 0.16 mmol) were dissolved in toluene (60 mL). After the flask was evacuated under vacuum and purged with dry nitrogen for three times, the mixture was refluxed at 120 ^{o}C for 12 h under nitrogen. After cooling to the room temperature, the reaction mixture was poured into water and extracted with dichloromethane three times. The combined organic layers were washed with water, and dried over anhydrous Na_2SO_4. After filtration and solvent evaporation, the residue was purified by silica-gel column chromatography using dichloromethane/petroleum ether as an eluent. The product was obtained as a yellowish green solid in 79% yield. ^{1}H NMR (500 MHz, DMSO-d_6, δ): 8.08 (d, J = 1.2 Hz, 1H), 8.06–8.01 (m, 3H), 8.00–7.95 (m, 1H), 7.82 (dd, J = 7.9, 1.5 Hz, 1H), 7.67–7.61 (m, 3H), 7.46–7.40 (m, 2H), 6.81–6.69 (m, 6H), 6.06–6.01 (m, 2H), 1.52 (s, 6H). ^{13}C NMR (125 MHz, DMSO-d_6, δ): 195.31, 155.00, 154.08, 143.80, 143.72, 142.59, 137.99, 137.72, 136.06, 133.88, 133.04, 131.07, 130.63, 129.30, 127.87, 124.28, 124.26, 123.55, 122.37, 121.79, 120.48, 115.97, 114.02, 47.24, 27.09. HRMS (MALDI-TOF): m/z [M^+] calcd. $C_{34}H_{25}NO_2$, 479.1885; found, 479.1896.

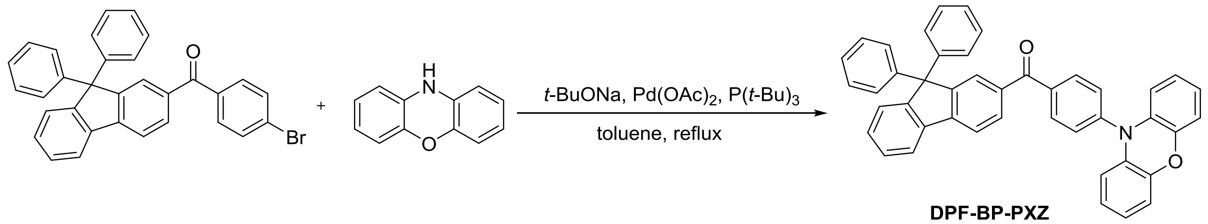

DPF-BP-PXZ

(4-(Phenoxazin-10-yl)phenyl)(9,9-diphenylfluoren-2-yl)methanone (DPF-BP-PXZ): The procedure was analogous to that described for DMF-BP-PXZ. Yellow solid, yield 85%. ^{1}H NMR (500 MHz, DMSO-d_6, δ): 8.18–8.13 (m, 1H), 8.09 (d, J = 7.5 Hz, 1H), 7.98–7.92 (m, 2H), 7.91–7.84 (m, 2H), 7.60–7.40 (m, 5H), 7.34–7.22 (m, 6H), 7.20–7.12 (m, 4H), 6.82–6.65

(m, 6H), 6.02–5.96 (m, 2H). ^{13}C NMR (125 MHz, DMSO-d_6, δ): 194.98, 152.11, 151.33, 145.39, 144.71, 143.70, 142.61, 138.77, 137.73, 136.36, 133.84, 133.02, 131.04, 130.99, 129.83, 129.06, 128.61, 128.07, 127.49, 126.79, 124.26, 122.38, 122.27, 121.06, 115.97, 113.94, 65.52. HRMS (MALDI-TOF): m/z [M^+] calcd. $C_{44}H_{29}NO_2$, 603.2198; found, 603.2177.

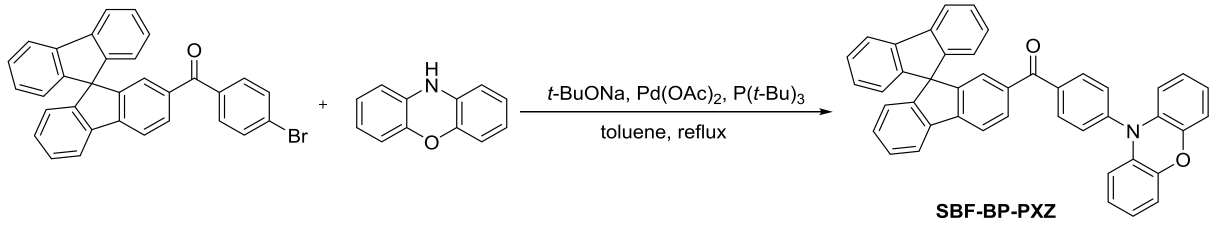

(4-(Phenoxazin-10-yl)phenyl)(9,9'-spirobi[fluoren]-2-yl)methanone (SBF-BP-PXZ): The procedure was analogous to that described for DMF-BP-PXZ. Yellow solid, yield 82%. ^{1}H NMR (500 MHz, DMSO-d_6, δ): 8.23 (d, J = 8.0 Hz, 1H), 8.18 (d, J = 7.6 Hz, 1H), 8.06 (d, J = 7.6 Hz, 2H), 7.91–7.83 (m, 3H), 7.56–7.41 (m, 5H), 7.30–7.23 (m, 1H), 7.21–7.14 (m, 2H), 7.06 (d, J = 1.2 Hz, 1H), 6.79–6.65 (m, 9H), 5.94 (dd, J = 7.7, 1.7 Hz, 2H). ^{13}C NMR (125 MHz, DMSO-d_6, δ): 194.77, 149.69, 149.47, 147.84, 146.40, 143.68, 142.49, 141.80, 140.48, 137.67, 136.38, 133.80, 132.81, 131.67, 130.95, 130.02, 128.87, 128.74, 128.70, 124.34, 124.25, 124.01, 122.36, 122.31, 121.21, 120.92, 115.95, 113.91, 65.87. HRMS (MALDI-TOF): m/z [M^+] calcd. $C_{44}H_{27}NO_2$, 601.2042; found, 601.2069.

3. X-ray crystallography

Crystal data for DMF-BP-PXZ (CCDC 1572670): $C_{34}H_{25}NO_2$, M_W = 479.55, orthorhombic, Pca21, a = 18.6113(11), b = 7.6173(4), c = 17.8937(12) Å, β = 90°, V = 2536.8(3) Å^3, Z = 4, Dc = 1.256 g cm^{-3}, μ = 0.077 mm^{-1} (MoKα, λ = 0.71073), F(000) = 1008, T = 173(2) K, 19327 measured reflections, 4548 independent reflections (Rint = 0.0785), GOF on F^2 = 1.023, R_1 = 0.0830, wR_2 = 0.0976 (all data).

4. OLED fabrication and characterization

Glass substrates pre-coated with a 95-nm-thin layer of indium tin oxide (ITO) with a sheet resistance of 20 Ω per square were thoroughly cleaned for 10 minutes in ultrasonic bath of acetone, isopropyl alcohol, detergent, deionized water, and isopropyl alcohol and then treated with O_2 plasma for 5 min in sequence. Organic layers were deposited onto the ITO-coated substrates by high-vacuum ($< 5 \times 10^{-4}$ Pa) thermal evaporation. Deposition rates were controlled by independent quartz crystal oscillators, which are 1~2 Å s^{-1} for organic materials, 0.1 Å s^{-1} for LiF, and 6 Å s^{-1} for Al, respectively. The emission area of the devices is 3 × 3 mm^{-2} as shaped by the overlapping area of the anode and cathode. All the device characterization steps were carried out at room temperature under ambient laboratory conditions without encapsulation except spectrum collection process. EL spectra were taken by an optical analyzer, Photo Research PR705. Current density and luminance versus driving voltage characteristics were measured by Keithley 2420 and Konica Minolta chromameter CS-200, respectively. External quantum efficiencies were calculated by assuming that the devices were Lambertian light sources.

5. Estimation of basic photophysical data

The quantum efficiencies and rate constants were determined using the following equations according to Adachi's method.

$$\Phi_{prompt} = \Phi_{PL} R_{prompt} \quad (1)$$

$$\Phi_{delayed} = \Phi_{PL} R_{delayed} \quad (2)$$

$$k_F = \Phi_{prompt}/\tau_{prompt} \quad (3)$$

$$\Phi_{PL} = k_F/(k_F + k_{IC}) \quad (4)$$

$$\Phi_{prompt} = k_F/(k_F + k_{IC} + k_{ISC}) \quad (5)$$

$$\Phi_{IC} = k_{IC}/(k_F + k_{IC} + k_{ISC}) \quad (6)$$

$$\Phi_{ISC} = k_{ISC}/(k_F + k_{IC} + k_{ISC}) = 1 - \Phi_{prompt} - \Phi_{IC} \quad (7)$$

$\Phi_{RISC} = \Phi_{delayed}/\Phi_{ISC}$ (8)

$k_{RISC} = (k_p k_d \Phi_{delayed})/(k_{ISC} \Phi_{prompt})$ (9)

$k_p = 1/\tau_{prompt}$; $k_d = 1/\tau_{delayed}$ (10)

6. Additional spectra and data

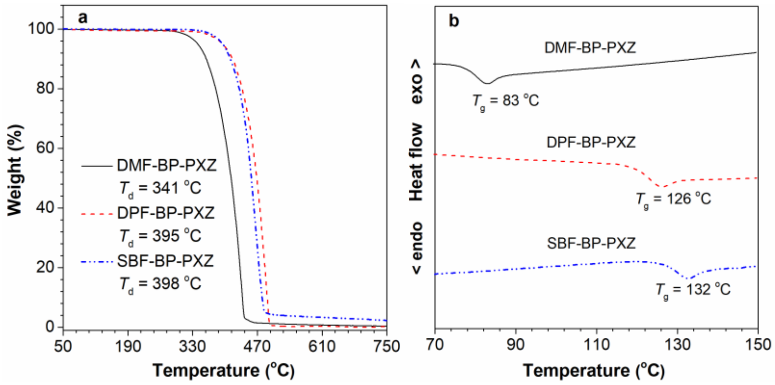

Figure S1. (a) Thermogravimetric analysis and (b) differential scanning calorimetry curves of DMF-BP-PXZ, DPF-BP-PXZ and SBF-BP-PXZ, recorded under nitrogen at a heating rate of 10 °C min^{-1}.

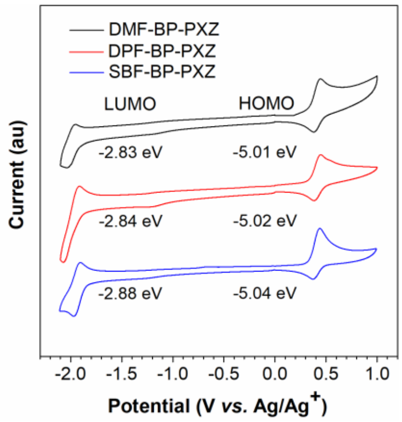

Figure S2. Cyclic voltammograms of the films of DMF-BP-PXZ, DPF-BP-PXZ and SBF-BP-PXZ, measured in acetonitrile containing 0.1 M tetra-*n*-butylammonium hexafluorophosphate. Scan rate: 100 mV s^{-1}.

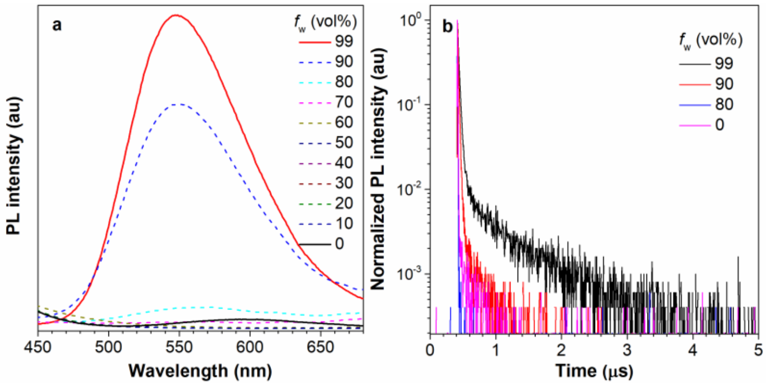

Figure S3. (a) PL spectra and (b) transient decay spectra of DPF-BP-PXZ in THF/water mixtures with different water fractions (f_w), measured under nitrogen.

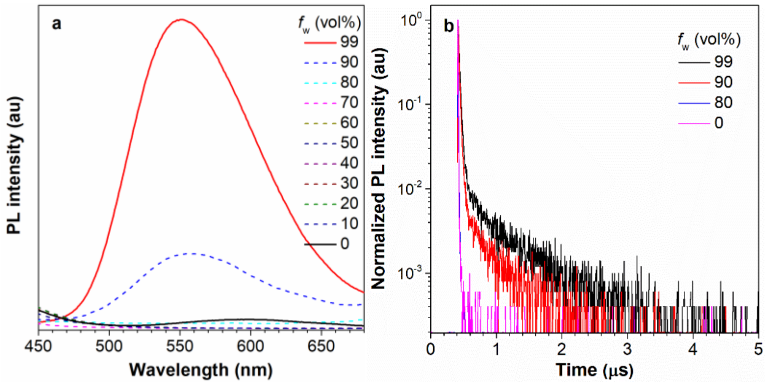

Figure S4. (a) PL spectra and (b) transient decay spectra of SBF-BP-PXZ in THF/water mixtures with different water fractions (f_w), measured under nitrogen.

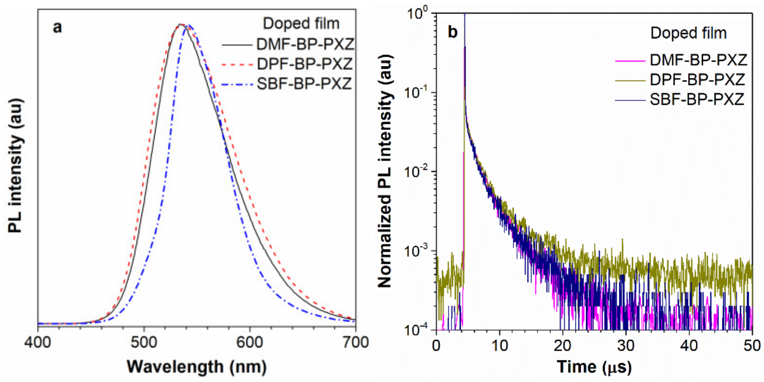

Figure S5. (a) PL spectra and (b) transient PL spectra of doped films (30 wt% in CBP) of DMF-BP-PXZ, DPF-BP-PXZ and SBF-BP-PXZ, measured at 300 K under nitrogen.

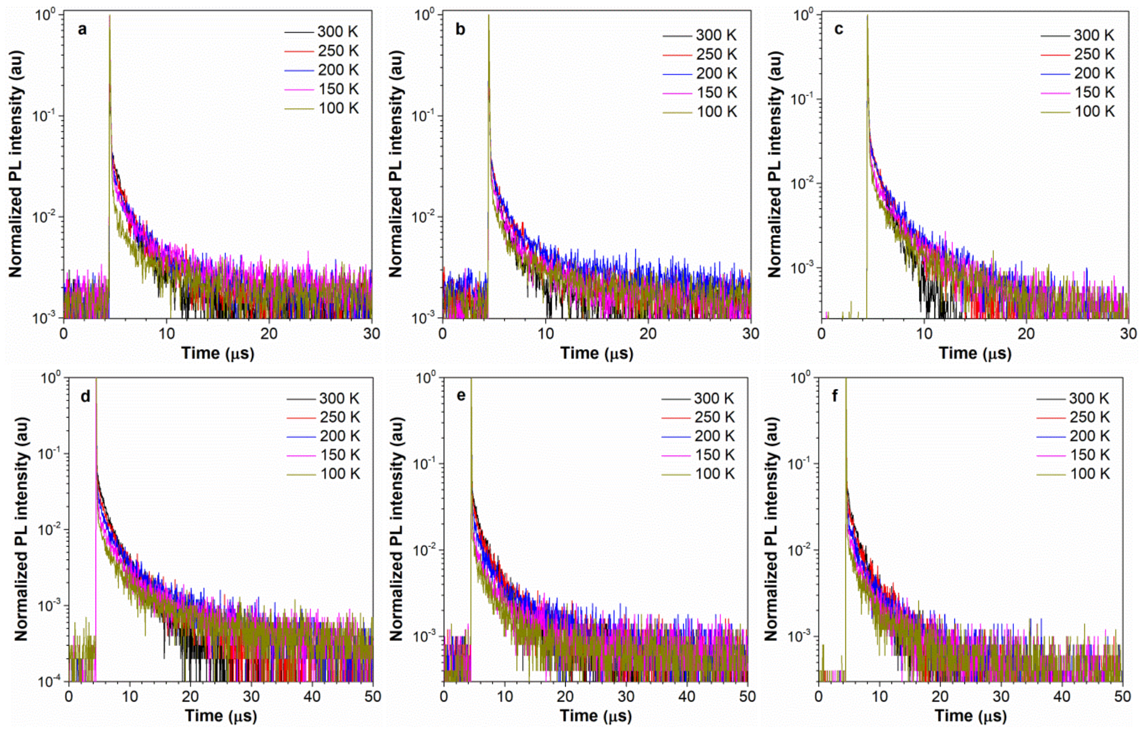

Figure S6. Temperature dependent PL transient decay spectra of (a) DMF-BP-PXZ, (b) DPF-BP-PXZ, and (c) SBF-BP-PXZ in neat films and (d) DMF-BP-PXZ, (e) DPF-BP-PXZ, and (f) SBF-BP-PXZ in doped films (30 wt % in CBP), measured under nitrogen.

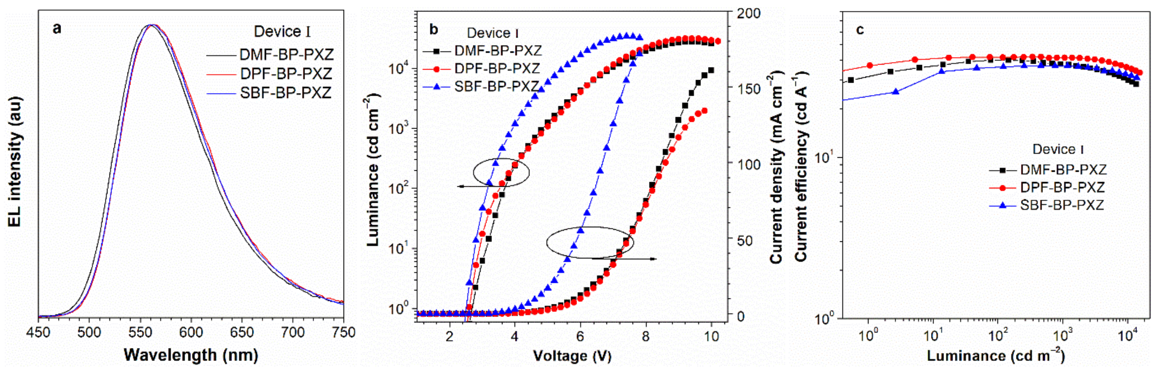

Figure S7. (a) EL spectra, (b) current density–voltage–luminance (J–V–L) characteristics, and (c) current efficiency versus luminance plots of the non-doped OLEDs.

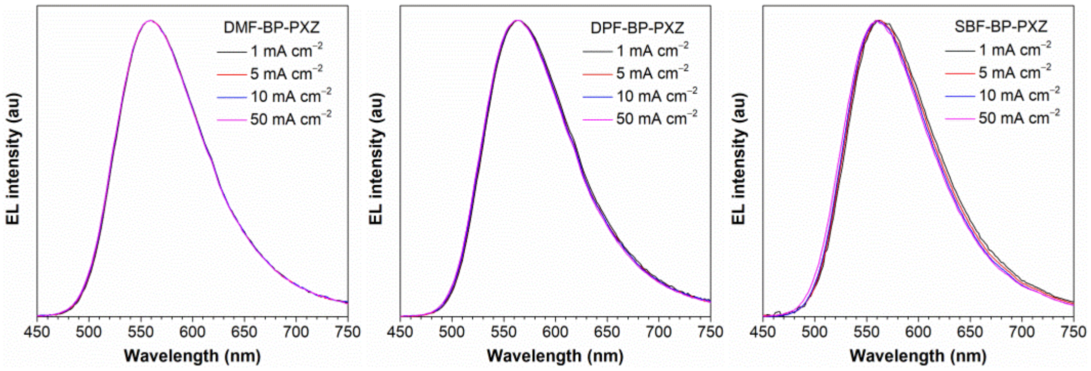

Figure S8. EL spectra of the non-doped OLEDs at different current densities.

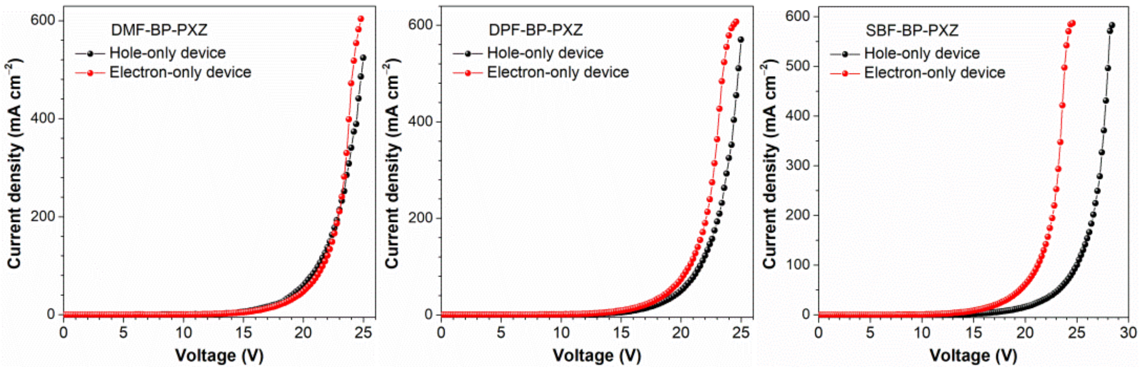

Figure S9. Current density-voltage characteristics of hole-only devices: ITO/TAPC (25 nm)/emitter (35 nm)/TAPC (55 nm)/Al, and electron-only devices: ITO/TmPyPB (25 nm)/emitter (35 nm)/TmPyPB (55 nm)/LiF (1 nm)/Al.

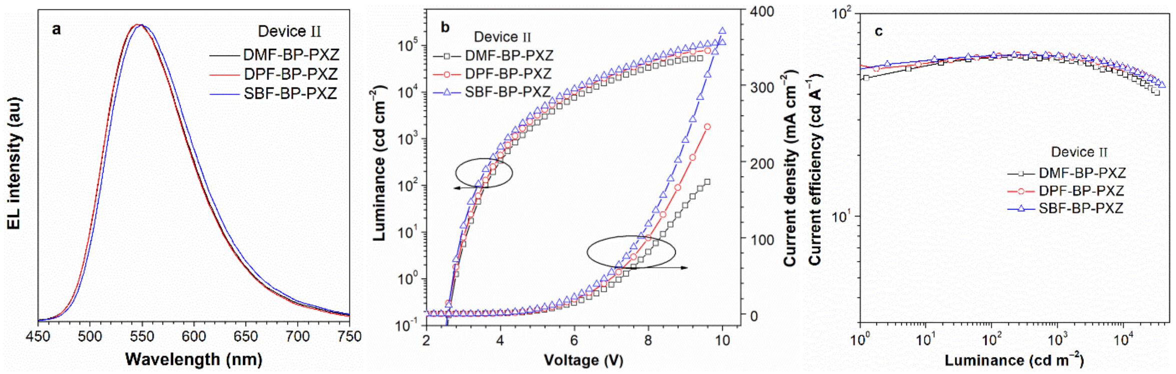

Figure S10. (a) EL spectra, (b) Current density–voltage–luminance (J–V–L) characteristics, and (c) Current efficiency versus luminance plots of the doped OLEDs.

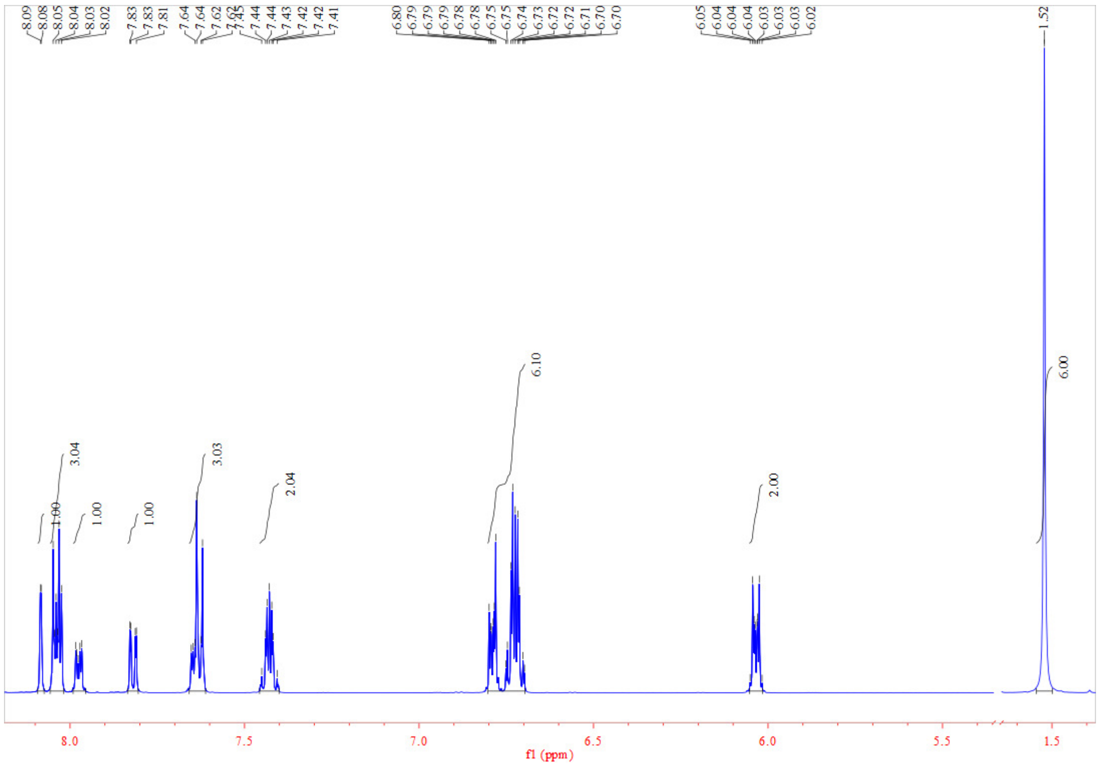

Figure S11. ^{1}H NMR spectrum of DMF-BP-PXZ in DMSO-d_6.

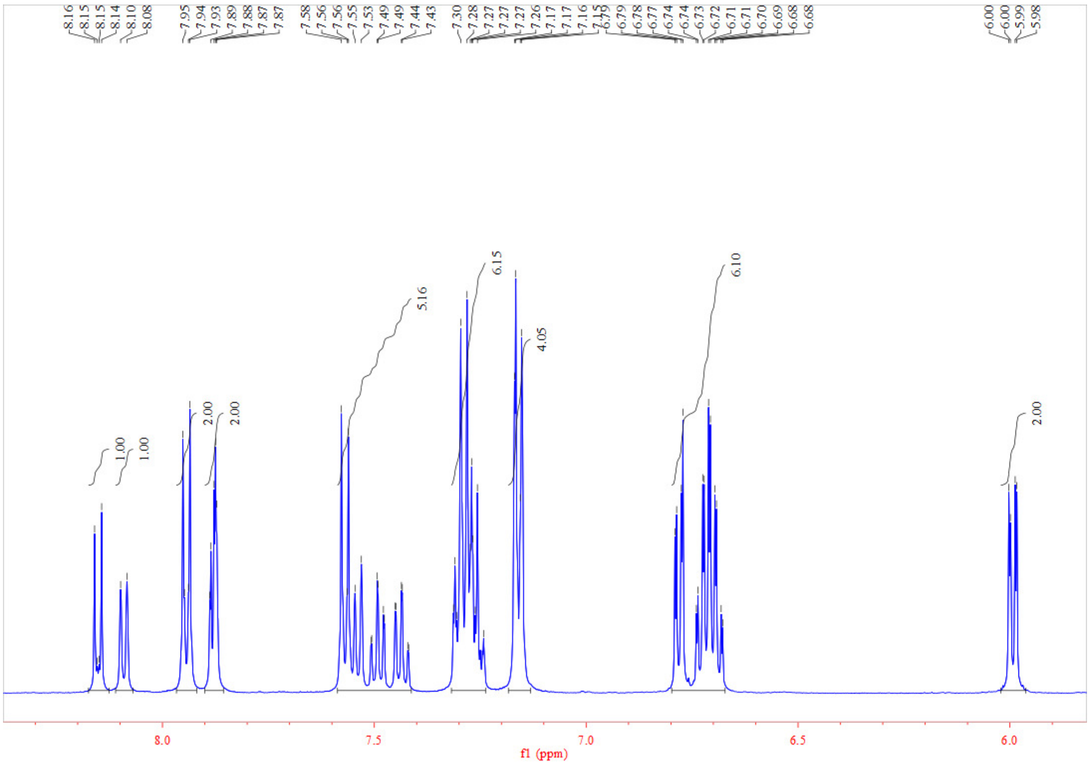

Figure S12. ^{1}H NMR spectrum of DPF-BP-PXZ in DMSO-d_6.

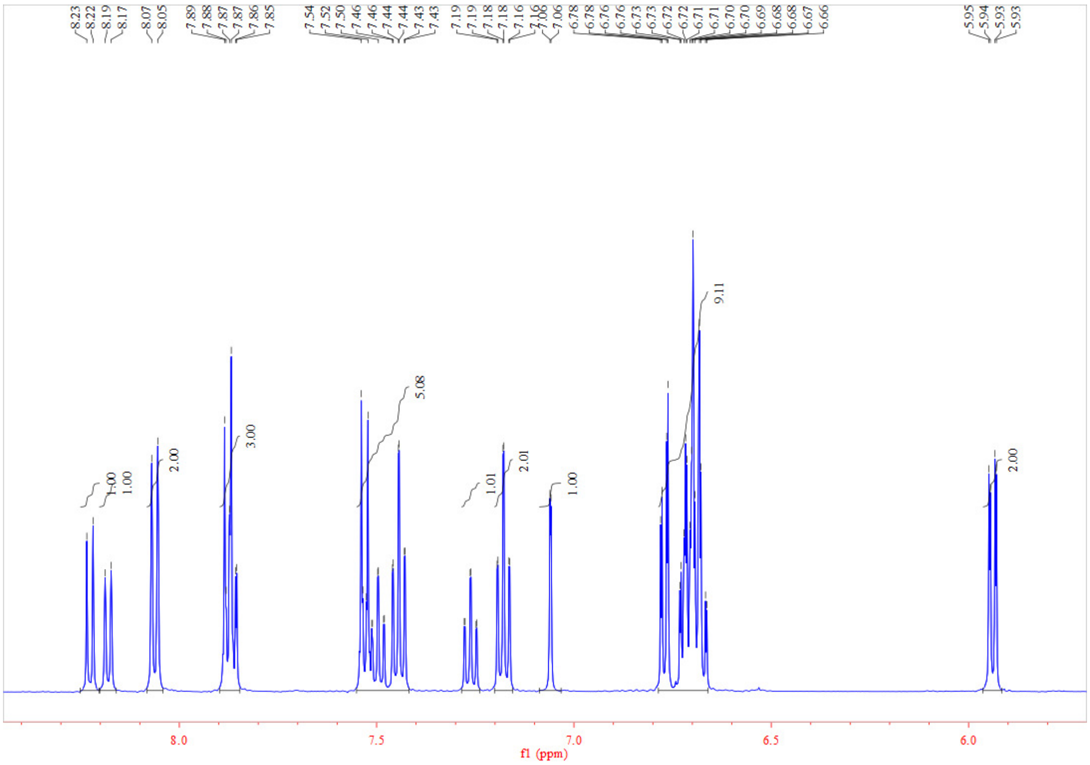

Figure S13. ^{1}H NMR spectrum of SBF-BP-PXZ in DMSO-d_6.

Table S1. Transient PL decay data of DMF-BP-PXZ, DPF-BP-PXZ and SBF-BP-PXZ in THF/water mixtures with different water fractions (f_w).

	f_w	$<\tau>$ (ns)	τ_1 (ns)	τ_2 (ns)	A_1	A_2	$R_{delayed}$ (%)
DMF-BP-PXZ	0	2.3	2.3	-	40609.2	-	-
	80	1.5	1.5	-	133208	-	-
	90	7.9	7.9	-	8408.8	-	-
	99	92.8	20.9	464.1	5604.6	49.0	16
DPF-BP-PXZ	0	3.2	3.2	-	22954.7	-	-
	80	1.8	1.8	-	72590.9	-	-
	90	15.9	11.7	64.5	7028.1	110.4	8
	99	132.0	22.7	574.9	5300.8	51.6	20
SBF-BP-PXZ	0	1.8	1.8	-	77084	-	-
	80	2.8	2.8	-	28753.2	-	-
	90	35.8	16.6	204.9	6275.7	57.7	10
	99	112.1	21.4	513.1	5761.7	54.3	18

Table S2. Emission wavelength (λ_{em}) and oscillator strength (f) from S_1 to S_0 for DMF-BP-PXZ are calculated in solid phase by different functionals.

Functional	λ_{em} (nm)	f
BMK	504	0.0006
M06	588	0.0004
M062X	453	0.0005
MPW1B95	573	0.0005
PBE33	537	0.0006
PBE38	503	0.0006
PBE0	620	0.0004

Table S3. Emission wavelength (λ_{em}) and Oscillator strength (f) from S_2 to S_0 for DMF-BP-PXZ are calculated in solid phase by MPW1B95 and PBE0.

Functional	λ_{em} (nm)	f
MPW1B95	494	0.0544
PBE0	525	0.0523

Table S4. Spin orbit coupling (SOC) values, reorganization energy (λ), singlet-triplet energy difference (ΔE) and intersystem crossing rates (k_{ISC}) at 300 K in THF.

	SOC (cm^{-1})	λ (meV)	ΔE (meV)	k_{ISC} (s^{-1})
$S_2 \rightarrow T_4$	0.135	956	–282	4.7×10^4
$S_2 \rightarrow T_3$	0.858	1109	–453	4.1×10^6
$S_2 \rightarrow T_2$	0.226	321	–600	2.2×10^6

Table S5. Spin orbit coupling (SOC) values, reorganization energy (λ), singlet-triplet energy difference (ΔE) and intersystem crossing rates (k_{ISC}) at 300 K in solid.

	SOC (cm^{-1})	λ (meV)	ΔE (meV)	k_{ISC} (s^{-1})
$S_2 \rightarrow T_3$	0.948	760	–223	6.6×10^6
$S_2 \rightarrow T_2$	0.270	45.3	–354	1.1×10^{-1}
$T_3 \rightarrow S_2$	0.948	263	223	7.2×10^4
$T_2 \rightarrow S_2$	0.270	310	354	3.8×10^1
